# Assessing the Readability of Online Keloid Resources

**DOI:** 10.7759/cureus.106538

**Published:** 2026-04-06

**Authors:** Kenechi Iwelumo, Katie Singh

**Affiliations:** 1 Dermatology, Rutgers Robert Wood Johnson Medical School, New Brunswick, USA

**Keywords:** dermatology, health literacy, keloid scars, online health information, patient education, readability, skin of color

## Abstract

Background: Keloids are fibroproliferative scars that can disproportionately affect individuals with skin of color. Given the complexity of treatment options, many patients seek guidance through online health resources. However, these materials may not be written at a level accessible to the general public, particularly those with limited health literacy.

Objective: To evaluate the readability of the top 100 Google search results for “keloid scar” and determine whether these online patient education materials (PEMs) meet the readability standards recommended by the American Medical Association (AMA).

Methods: A Google search was conducted on April 29, 2025, using incognito mode with cleared cookies to minimize bias. The first 100 websites were screened, with exclusions for duplicate content, insufficient text (<250 words), scientific articles, clinician-targeted pages, and non-educational material. A total of 40 websites met the inclusion criteria. Readability was assessed using six validated formulas: Flesch-Kincaid Grade Level, Simple Measure of Gobbledygook (SMOG), Gunning Fog, Coleman-Liau, Automated Readability Index, and Linsear Grade Level. Mean grade-level scores were calculated and stratified by source type.

Results: Only 12.5% (5/40) of keloid-related PEMs had an average grade-level score at or below the AMA’s recommended sixth-grade threshold. The average readability across all materials was at the 10th-grade level (mean=9.54; range 6.00-12.84). When considering individual formulas, the highest reading level assigned per resource ranged from grade 11.09 to 15.02. Government websites had the lowest mean readability (sixth-grade level), while “Other” sources scored highest (mean=10.38). Academic/hospital-based sites (mean=9.26), commercial (mean=9.79), and non-profit sources (mean=9.53) also exceeded recommended levels. A one-way analysis of variance (ANOVA) showed no significant difference in readability across source types (p=0.122).

Limitations: Only English-language, text-based materials were analyzed; multimedia content and regional search variations were not assessed.

Conclusions: Online PEMs related to keloid scars exceed recommended readability levels, potentially limiting their utility for patients with low health literacy. These findings underscore the need for more accessible, culturally inclusive materials, especially for populations disproportionately affected by keloids.

## Introduction

Keloids are classified as fibroproliferative disorders that result from abnormal wound healing following skin trauma or inflammation [1]. This condition has a higher prevalence among individuals with more pigmented skin [2]. While counseling by a treating physician remains central to patient education, many patients also turn to online resources to better understand their condition and available treatment options. Given that keloids are often challenging to manage and may require long-term or multimodal therapy, accessible and comprehensible online information can play an important supportive role in reinforcing physician guidance, improving patient awareness of available treatments, and facilitating more informed discussions during clinical encounters. In this context, inadequate or unclear information may contribute to misconceptions, delayed care-seeking, or reduced engagement with recommended therapies.

Health literacy is a significant determinant of an individual’s ability to process medical information and engage in shared decision-making [3,4]. Unfortunately, lower health literacy is common in communities affected by systemic barriers to healthcare access [5]. This further underscores the need for patient education materials (PEMs) that accommodate varying literacy levels. The American Medical Association (AMA) recommends that PEMs be written at or below a sixth-grade reading level to ensure accessibility to a broad audience [6]. However, existing studies have shown that many materials surpass this benchmark, diminishing their usefulness for patients with limited literacy skills [7].

Readability formulas offer standardized approaches for assessing the complexity of written content. Common indices include the Flesch-Kincaid Grade Level, Simple Measure of Gobbledygook (SMOG) Index, Gunning Fog Index, Coleman-Liau Index, and Automated Readability Index, each incorporating distinct textual elements [8-11].

Despite the prevalence of keloids and documented disparities in access to health information, few studies have assessed the readability of online PEMs specific to this condition. This study aims to evaluate the readability of the top 100 Google search results for “keloid scar” and determine whether these materials conform to the established guidelines for health communication.

## Materials and methods

Search strategy and website selection

**Figure 1:**
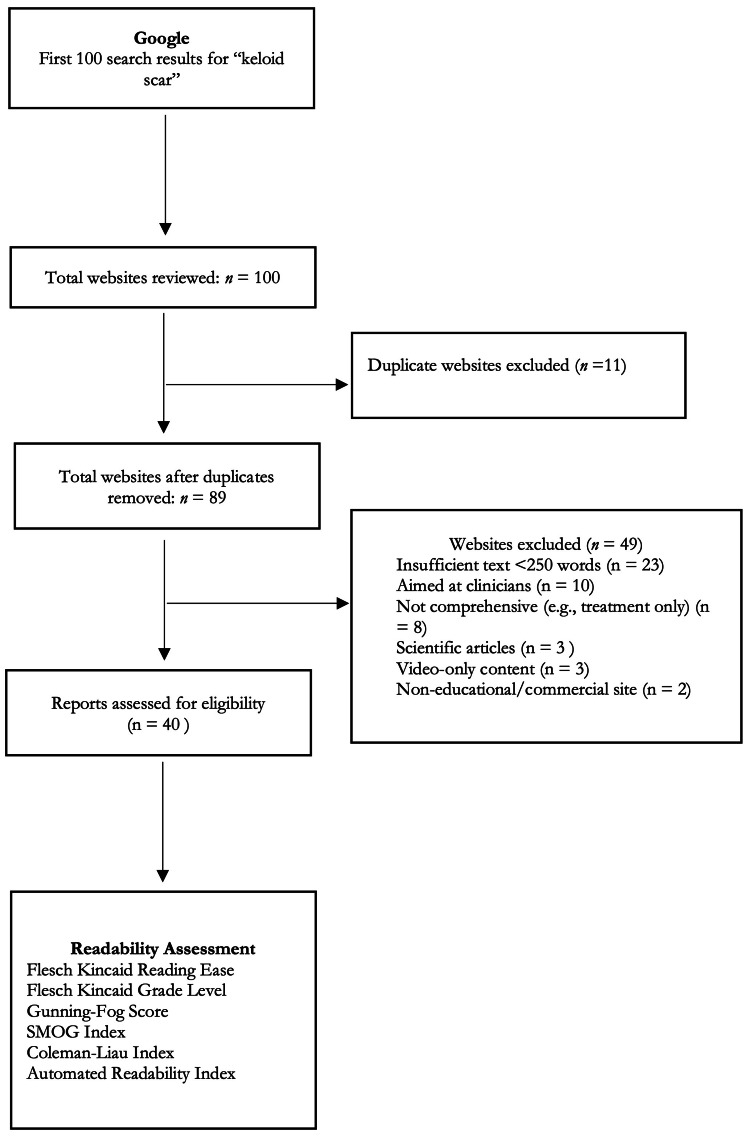
Schematic depicting the identification and screening of top-searched online patient health resources on keloid scars SMOG: Simple Measure of Gobbledygook

A Google search using the term “keloid scar” was conducted on April 29, 2025. To minimize bias from location and prior searches, cookies and cache were cleared, and the search was performed in incognito mode. The search yielded approximately 17,100,000 results, from which the first 100 websites were screened. This approach mirrors prior studies evaluating the quality and accessibility of online health information [12,13]. Inclusion criteria included materials written in English, patient-facing educational materials, and websites with “keloid” in the title. Duplicate websites were removed (n=11), leaving 89 unique websites. Further exclusion criteria included insufficient textual content (<250 words), clinicians as the target audience, non-comprehensive content (e.g., treatment-only pages), scientific articles, video-only content, and commercial content. Ultimately, 40 websites met eligibility criteria and were used for analysis (Figure 1).

Readability analysis

Each eligible website was evaluated using the Readability Formulas online calculator (https://readabilityformulas.com) [14]. Six validated readability formulas were applied: Flesch-Kincaid Grade Level, SMOG, Gunning Fog, Coleman-Liau, Automated Readability Index, and Linsear Grade Level (Table 1). A mean grade-level score was computed for each source. Materials were also categorized by source type (e.g., academic, government, non-profit, commercial) to assess potential differences in readability. Websites belonging to private dermatology or cosmetic practices were included in the “Other” category due to variability in content scope, lack of standardized authorship, and small sample size. These sites often served a dual purpose of education and self-promotion, making them distinct from academic or governmental resources but not uniformly commercial in nature.

**Table 1:** Commonly used readability formulas in healthcare † indicates whether the formula provides a U.S. grade-level equivalent; if not, the score is numerical and can be converted to a grade level. Sources for readability formulas include: Automated Readability Index [8], Flesch Reading Ease and Flesch-Kincaid Grade Level (FKGL) [8,9], Gunning Fog Index [8,10], Coleman-Liau Index [10], Simple Measure of Gobbledygook (SMOG) [9,11], and Linsear Write and Linsear Grade Level [10]

Index	Formula Components	Grade-Level Output^†^	Strengths	Limitations
Automated Readability Index	Characters per word, sentence length	Yes	Useful for automated systems (uses characters)	Overestimates complexity in health materials
Flesch-Kincaid	Average sentence length, average syllables per word	Yes	Commonly used, easy to interpret	May underestimate the reading grade level
Flesch Reading Ease	Same as FKGL but outputs a score (0-100)	No	Used in popular word processor applications	Results reported as broad ranges over eighth-grade level
Gunning Fog	Sentence length, complex words (≥3 syllables)	Yes	Useful for business and technical contexts	May overestimate the difficulty of long, commonly understood words
Coleman-Liau	Characters per word, sentence length	Yes	Useful for automated systems (uses characters)	May overestimate the difficulty of long, commonly understood words
SMOG	Number of sentences, complex words (≥3 syllables)	Yes	Designed for health content, validated for public education	Sensitive to the size of the text sample
Linsear Write (Original)	Easy words (1-2 syllables), hard words (3+ syllables), sentence length, sentence count	No	Formula is straightforward, and can be calculated manually	Shorter text may be inaccurate, originally designed for longer passages
Linsear Grade Level	Adjusted from the original formula to yield the U.S. grade level	Yes	Used in military and technical training settings	-

## Results

Among the 40 selected PEMs on keloids, readability scores varied across the six formulas analyzed. The average readability across all materials was approximately at the 10th-grade level (mean=9.54, range=6.00-12.84), exceeding the AMA’s recommended sixth-grade reading level by nearly four grade levels. Notably, only 12.5% (5 out of 40) of resources had an average grade level.

**Figure 2:**
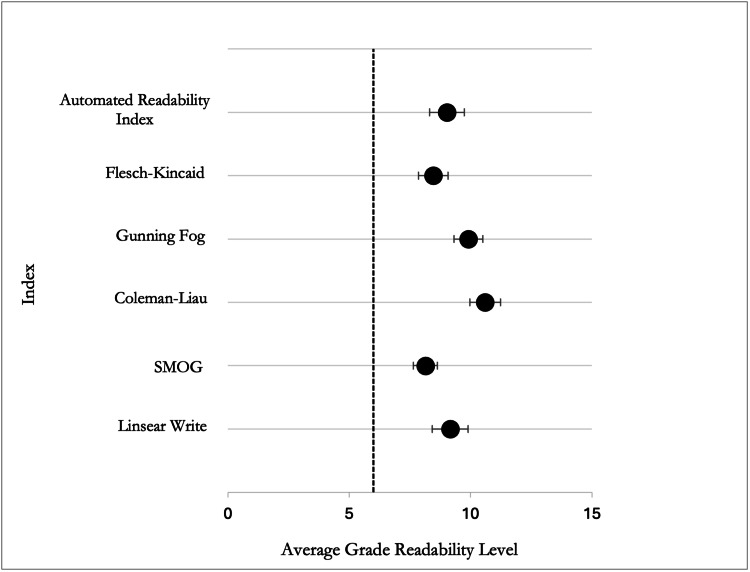
Average readability grade level by index compared with the AMA’s recommended sixth-grade reading level AMA: American Medical Association; SMOG: Simple Measure of Gobbledygook

When examining individual readability formulas, the highest reading level assigned to each resource ranged from grade 11.09 to 15.02, suggesting that even materials with an acceptable average may contain sections that present significant comprehension challenges, depending on the formula used. All six readability indices produced average scores that were 2.1 to 4.6 grade levels above the recommended sixth-grade level (Figure 2).

**Figure 3:**
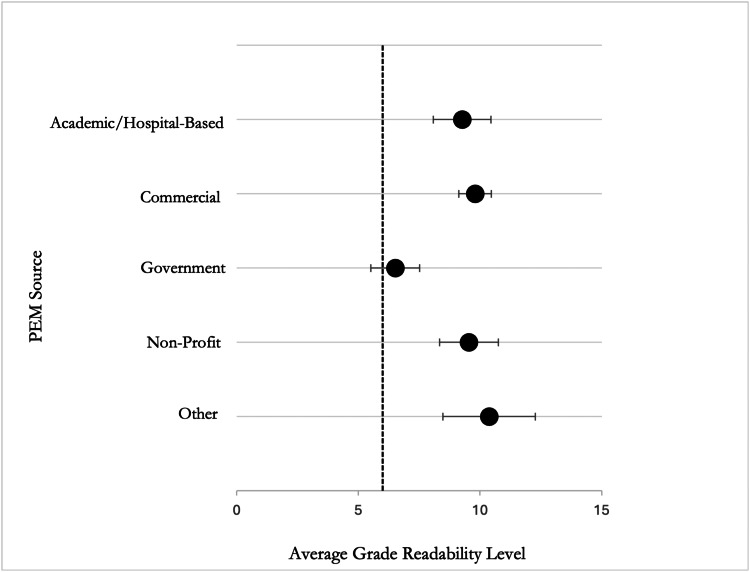
Average readability grade level by source type compared with the AMA’s recommended sixth-grade reading level AMA: American Medical Association; PEM: patient education material

Readability scores also varied by source type. Materials from government websites (n=2) yielded the lowest mean readability score (sixth-grade level), while websites classified as “Other” (n=6) had the highest (mean=10.38). Academic/hospital-based sources (n=9, mean=9.26), commercial websites (n=13, mean=9.79), and non-profit organizations (n=10, mean=9.53) also exceeded the recommended level. However, a one-way analysis of variance (ANOVA) revealed no statistically significant difference in mean readability scores across source types (p=0.122; Figure 3).

Limitations of this study include the use of only English-language websites and the exclusion of non-textual and multimedia content, which may enhance patient comprehension.

## Discussion

This study demonstrates a persistent and clinically meaningful gap between recommended readability standards and the actual complexity of online PEMs addressing keloid scars. Despite longstanding guidance from the AMA that health materials be written at or below a sixth-grade reading level, the overwhelming majority of resources analyzed exceeded this benchmark. With a mean readability approaching the 10th-grade level, these materials may be inaccessible to a substantial portion of the population, particularly individuals with limited health literacy.

Our findings are consistent with prior studies evaluating general online health content, which have similarly reported that patient-facing materials frequently surpass recommended readability thresholds [7]. Although prior investigations have evaluated readability in conditions with keloid-like clinical features, such as acne keloidalis nuchae (AKN), these entities are pathophysiologically distinct from true keloids. The present study focuses specifically on keloids, thereby capturing a broader spectrum of patient concerns beyond a single, anatomically localized condition [15]. By doing so, it captures a wider spectrum of patient concerns, including cosmetic, psychosocial, and culturally relevant considerations that extend beyond a single clinical subtype.

These findings align with broader literature demonstrating that dermatologic and general medical online resources frequently surpass recommended readability thresholds. However, keloids represent a uniquely important condition in this context. Unlike many dermatologic disorders, keloids disproportionately affect individuals with skin of color. These populations are more likely to experience structural barriers to care, including reduced access to specialty services, lower health literacy rates, and historical underrepresentation in medical research and educational materials. When educational resources are written at reading levels that exceed national recommendations, they may unintentionally exacerbate existing disparities.

Keloids are also notoriously challenging to treat, often requiring multimodal therapy, repeated clinical visits, and long-term adherence to interventions such as intralesional corticosteroids, laser resurfacing treatments, or surgical excision with adjuvant therapy. Treatment decisions frequently involve nuanced discussions regarding recurrence risk, cosmetic outcomes, and potential adverse effects. Inadequate comprehension of these complexities may contribute to unrealistic expectations, poor adherence, or premature discontinuation of therapy. Thus, readability is not merely a theoretical concern but one that may directly influence clinical outcomes.

Interestingly, although government websites demonstrated the lowest mean readability scores, no statistically significant differences were observed across source types. This suggests that readability challenges are widespread rather than isolated to specific sectors. Even academic and hospital-based websites, which are often perceived as authoritative and patient-centered, exceeded recommended levels. Private practice and hybrid promotional-educational websites in the “Other” category demonstrated the highest complexity, potentially reflecting marketing language or unstandardized authorship practices. These findings underscore the need for systematic readability auditing across all content-producing entities.

Future efforts should prioritize plain-language revisions of existing PEMs, incorporation of community input during content development, and routine readability testing prior to publication. Strategies such as shorter sentences, substitution of complex medical terminology with everyday language, use of bullet points, and inclusion of glossaries can substantially lower reading levels without sacrificing accuracy. Additionally, collaboration with patients from disproportionately affected communities may enhance both accessibility and trustworthiness.

Ultimately, improving readability in keloid-related educational materials represents a tangible and achievable step toward advancing health equity in dermatology. By aligning online resources with established literacy standards and cultural considerations, clinicians and institutions can better support informed decision-making, strengthen therapeutic partnerships, and empower patients navigating this often distressing and chronic condition.

## Conclusions

Online resources addressing keloids fail to meet established readability standards, limiting their utility for populations with low health literacy. Efforts to improve PEMs should prioritize clarity, accessibility for underserved populations, and alignment with AMA-recommended readability levels. Future work may benefit from incorporating patient feedback in the design of PEMs to ensure accessibility and relevance. Improving readability has the potential to enhance shared decision-making, promote treatment adherence, and reduce misinformation in communities disproportionately affected by keloids. Ultimately, greater attention to health literacy in dermatology can help bridge persistent disparities and empower patients to engage more effectively in their care.
